# Syringobulbia in Patients with Chiari Malformation Type I: A Systematic Review

**DOI:** 10.1155/2019/4829102

**Published:** 2019-03-19

**Authors:** Jian Shen, Jie Shen, Kaiyuan Huang, Yixin Wu, Jianwei Pan, Renya Zhan

**Affiliations:** ^1^Department of Neurosurgery, First Affiliated Hospital, School of Medicine, Zhejiang University, China; ^2^Cancer Hospital of Zhejiang, China

## Abstract

This study aimed to summarize the clinical features, diagnosis, and treatment of Chiari malformation type I- (CM-1-) associated syringobulbia. We performed a literature review of CM-1-associated syringobulbia in PubMed, Ovid MEDLINE, and Web of Science databases. Our concerns were the clinical features, radiologic presentations, treatment therapies, and prognoses of CM-1-associated syringobulbia. This review identified 23 articles with 53 cases. Symptoms included headache, neck pain, cranial nerve palsy, limb weakness/dysesthesia, Horner syndrome, ataxia, and respiratory disorders. The most frequently involved area was the medulla. Most of the patients also had syringomyelia. Surgical procedures performed included posterior fossa decompression, foramen magnum decompression, cervical laminectomy, duraplasty, and syringobulbic cavity shunt. Most patients experienced symptom alleviation or resolution postoperatively. A syringobulbic cavity shunt provided good results in refractory cases. Physicians should be aware of the possibility of syringobulbia in CM-1 patients, especially those with symptoms of sudden-onset brain-stem involvement. The diagnosis relies on the disorder's specific symptomatology and magnetic resonance imaging. Our review suggests that the initial therapy should be posterior fossa decomposition with or without duraplasty. In refractory cases, additional syringobulbic cavity shunt is the preferred option.

## 1. Introduction

Syringobulbia (SB) refers to a longitudinally oriented fluid-filled cavity within the brain stem. It is intimately associated with syringomyelia (SM), which is more common [1–3]. Many conditions can cause syringobulbia, including posterior fossa or spinal cord neoplasms, inflammatory disorders (e.g., arachnoiditis and meningitis), and Chiari malformations [1, 4]. SM occurs in CM-I patients at an incidence of 80%, [5] whereas case reports of CM-1-associated SB are rare. Studies of each disorder, however, have only a limited number of patients. Menezes et al. recently reported a series of 326 pediatric CM-I patients, with only 13 (4%) identified as having secondary SB [6]. Because of the rarity of SB, its manifestations, treatment methods, and long-term prognosis are still not established. We therefore conducted a systematic review to clarify the clinical characteristics of these patients.

## 2. Literature Search Strategy

This systematic review was conducted according to PRISMA (Preferred Reporting Items for Systematic Reviews and Meta-Analyses) guidelines and recommendations. Two reviewers (J.S., K.Y.H.) independently performed a literature search. PubMed, Ovid MEDLINE, and Web of Science databases were searched from their dates of inception to August 2018 using combinations of the following terms: “Arnold–Chiari malformation/Chiari malformation,” “type 1,” “tonsillar herniation,” “syringobulbia,” “syringomyelia,” and “syringitis/syrinx.” The reference lists of all retrieved articles were reviewed for identification of further potentially relevant studies.

### 2.1. Selection Criteria

Eligible case reports were systematically assessed using the following inclusion criteria: (1) diagnosis of CM-I with syringobulbia confirmed by magnetic resonance imaging (MRI) and (2) case reports and/or case series. Exclusion criteria were (1) presence of syringobulbia secondary to other causes (e.g., trauma, tumor, and shunt device malfunction) and (2) no syringobulbia identified on MRI. All publications were limited to those involving human subjects and those written in the English language. Editorials and expert opinions were excluded. Review articles were omitted because of potential publication bias and duplication of results.

### 2.2. Data Extraction and Critical Appraisal

The data were extracted from article texts, tables, and figures, with any estimates made based on the presented data and figures. If more information was needed for clarification, we attempted to contact the study's authors. Two investigators independently reviewed each retrieved article (K.Y.H., J.S.).

## 3. Results

### 3.1. Literature Search

**Figure 1:**
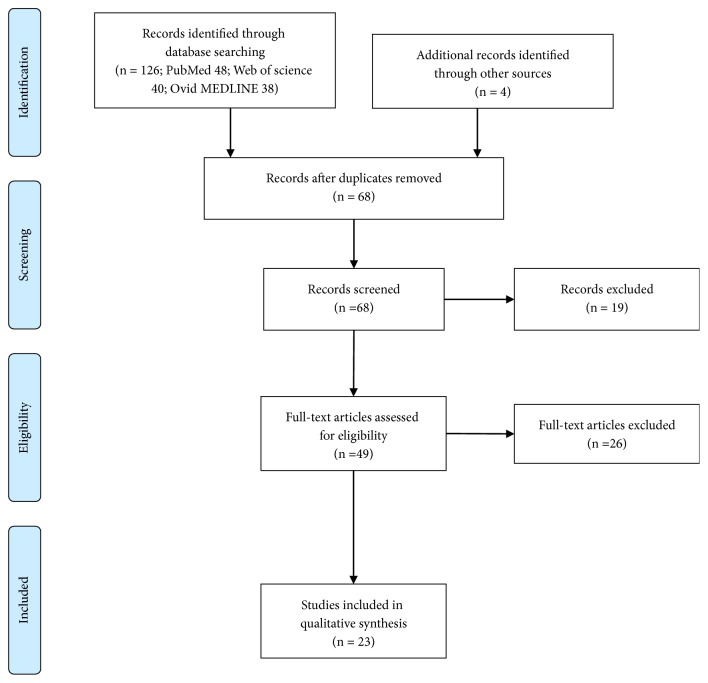
Flow diagram of the literature review.

The search strategy identified 130 studies. After removing 62 duplicate studies, our inclusion and exclusion criteria were applied to the titles of the 68 remaining articles, which yielded 49 studies that underwent full-text analysis. Finally, 23 articles with 53 cases were included in our study (Figure 1).  Table 1 and the Supplementary Table (available here) detail the clinical presentations, operative therapies, and outcomes for each patient (three case series with 30 patients are presented in the Supplementary Materials) [1, 5, 6].

### 3.2. Clinical Presentation

The symptoms of syringobulbia included headache, neck pain, cranial nerve palsy, limb weakness or dysesthesia, Horner syndrome, ataxia, and respiratory disorders. Limb weakness, whether unilateral or bilateral, was the most common symptom. Cranial nerves (CNs) IX and X were most frequently involved, manifesting as bulbar palsy, dysphagia, and dysarthria. Diplopia and ptosis were also common. Neurological examinations often revealed tendon hyperreflexia, although hyporeflexia sometimes occurred. Ataxia, gait disturbance, eye nystagmus, and other signs of cerebellum involvement were also common. Dyspnea and respiratory arrest, with an incidence of 9.4% (5/53), were unique presentations of medulla involvement. Other rare presentations included trismus, persistent singultus, and oscillopsia.

### 3.3. Imaging Findings

Syringobulbia was identified on MRI in all but one patient [7]. The medulla was involved in all patients, and solo medulla syringobulbia was most common. Pons involvement was also frequent, whereas the cerebrum was involved in only three patients. Syringobulbia usually presents on MRI as a silt-like lesion in one side of the medulla. Most patients had combined syringomyelia, which could be cervical, cervicothoracic, or holochord. Only five patients did not have syringomyelia (9.4%).

### 3.4. Surgical Treatment and Outcomes

**Figure 2:**
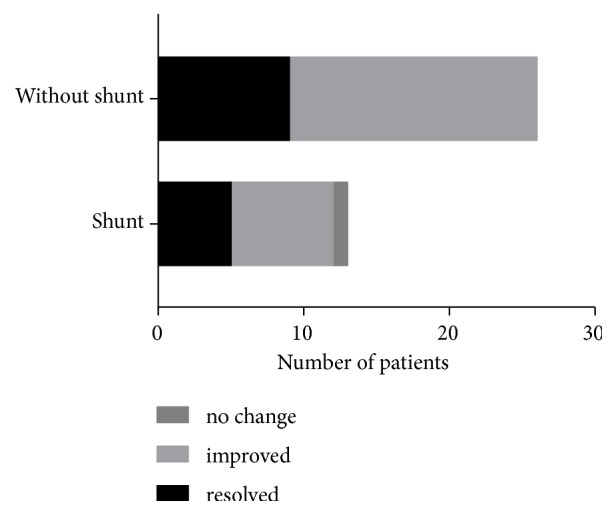
Surgical outcomes with and without the syringobulbic cavity shunt to the subarachnoid space.

Surgical procedures included posterior fossa (or suboccipital) decompression, foramen magnum decompression, cervical laminectomy, duraplasty, and syringobulbic cavity shunt. SB was alleviated in most cases. In refractory cases, a syringobulbic cavity shunt to the subarachnoid space was mostly successful. The ratio of complete resolution of symptoms increased among patients who underwent the shunt (Figure 2), although this result lacks statistical support because of the quality of the data.

## 4. Discussion

CM-1 is a common neurosurgical condition in both pediatric and adult populations. According to the literature, the rate of accompanying syringomyelia could be as high as 80%, whereas accompanying syringobulbia is much rarer. Menezes et al. reported that the incidence of syringobulbia was 4% in a series of 326 pediatric patients with CM-I [6]. Our literature review identified 23 cases with 130 CM-I-associated syringobulbia patients.

Although the symptomatology of CM-I has been well described in the literature, patients with accompanying syringobulbia have more prominent symptomatology because of the brain-stem involvement. SB-related symptoms usually appear in a chronic pattern, although sudden-onset cases have occurred, especially when there is a sudden onset of CN X palsy [8–10]. Headache, gait and balance disorders, and limb weakness or dysesthesia were the most frequent complaints of CM-I patients with SB. Cranial nerve dysfunction was common [11, 12]. CNs IX and X were most frequently affected, followed by CN V [13]. Persistent singultus that lasted >48 h, oscillopsia, nystagmus, and Horner syndrome were revealing presentations of syringobulbia [13–15]. Horner syndrome was present in 18% of patients in a previous study [16–18]. Central hypoventilation syndrome also occurred in CM-I patients with syringobulbia and, in fact, is the leading cause of sudden death in patients with CM-I [19, 20].

MRI was the most useful technique for detecting syringobulbic cavities [21]. A slit-like hyperintense area in the medulla was the characteristic presentation, [3, 6, 22] although in some patients the silt-like cavity was too thin to be visualized [7]. Other studies noted that SB could appear in three locations: the floor of the fourth ventricle, the midline floor of the fourth ventricle, or areas ventral to the fourth ventricle [15, 23]. The medulla is most frequently involved and was reported in all cases of this review. Further rostral extension into the pons (syringopontia), midbrain (syringomesencephaly), or cerebrum (syringocephaly) also occurred in several cases [24]. Although the cavity of syringobulbia is usually unilateral, midline cavities also occurred in some patients, leading to bilateral neurological defects.

The mechanism of syringobulbia formation is not clear. Tubbs et al. reported that there were no morphometric peculiarities for patients with CM-I-associated syringobulbia in regard to other CM-I patients with and without isolated syringomyelia [25]. Sherman et al., [21] in an MRI study, found that there are two types of syringobulbia. One type presents with thin clefts or slits extending into the medulla, with much smaller cavities than cervical cavities. The other type presents with saccular syringobulbia, with isometric medullary cavities, unlike the cervical or spinal syrinx cavity. Most cases are of the first type. Considering the close relation of syringobulbia and syringomyelia, syringobulbia might result from upward impulsive fluid movements due to previously established syringomyelia [26]. In contrast, the second type could be congenital—that is, the syringobulbia occurred during the fetal stage. Syringobulbia clefts due to dissection of cerebrospinal fluid (CSF) under pressure from the fourth ventricle should be differentiated from the ascending syringobulbia [16]. Other studies found that arachnoid veils or arachnoidal scars could cause syringobulbia or syringomyelia by partially obstructing CSF flow, which is often observed during surgery [6, 27]. Williams et al., however, thought that the most common correlation of syringobulbia was with none of the above mechanisms but with pressure differences acting downward on the hindbrain, with distortion of the cerebellum and stem, traction on the cranial nerves, and/or indentation of the brain stem by vascular loops [16]. The dramatic alleviation of SB and SM following posterior fossa decompression indicates a pathogenetic role of the tonsils or intradural pathology at the level of the egress of the fourth ventricle [6, 27].

Syringobulbia progression is usually slow [28]. The main surgical therapy is laminectomy for syringomyelia and posterior fossa or foramen magnum decompression for CM-I [6, 22]. Whether post-decompression duraplasty improves the outcome is still controversial. Recent meta-analyses supported reconstructive duraplasty in addition to compression because the overall clinical improvement is better [29–31]. In most cases, posterior fossa decompression with intradural exploration and duraplasty leads to its resolution [6]. Several reports suggested that a syringobulbia cavity–fourth ventricle shunt or syringobulbia cavity–subarachnoid space shunt benefits resolution [6, 7]. If the syringobulbia is communicating with the fourth ventricle, a fourth ventricle opening into the subarachnoid space is needed [7, 32].

## 5. Conclusion

Syringobulbia, a rare entity closely associated with CM-I, is characterized by an abrupt onset of symptoms due to the brain-stem involvement. The diagnosis mainly relies on the unique symptomatology of syringobulbia and MRI. Treatment is posterior fossa decomposition with or without duraplasty. An additional syringobulbic cavity shunt may improve the rate of total resolution of symptoms, especially in refractory cases.

## Figures and Tables

**Table 1:** Summary of CM-I patients with syringobulbia.

Author, year	Age, sex	Manifestations	Examination findings	Extent of SB	Extent of SM	Operation	Outcome	Follow-up
Feinberg M., 2016	3, M	Trismus	Completely normal aside from isolated trismus	Medulla, right pontine tegmentum	C1-T8	Suboccipital craniectomy, C-1 laminectomy	Resolved	1yr
Shankar, B., 2014	22, M	Persistent singultus	Ataxia, dysesthesia	Medulla	Holocord	Decompression	Resolved	None
Massimi, L., 2011	38, M	Acute respiratory failure	Consciousness, normal ICP	Medulla	Hydrocephalus, holocord	ETV	Resolved	3yrs
	1, M	Hemiparesis, Horner syndrome	Left flaccid hemiparesis, myosis, ptosis, Horner syndrome	Medulla, pons	Cervicothoracic	PFD, C-1 laminectomy, and duraplasty	Improved	3yrs
	2.5, M	Tetraparesis, dyspnea, neck pain	Tetraplegia, limb and trunk hypesthesia	Medulla, pons	Cervicothoracic	Not mentioned	Improved	10m
Massey, S. L., 2011	4, F	Eye inward deviation, neck tilting	CN VI, VII palsies, ataxia, limb spasticity	Medulla, pons	Cervicothoracic	Decompression	Alleviated	1.5yrs
Viswanatha, B., 2009	36, F	Dysarthria, headache, neck pain	CN IX, X palsies, dysarthria	Medulla, pons	Holocord	FMD	Improved	1yr
Robert E., 2009	16, M	Tetraparesis, headaches, urinary retention	CN VI palsies, tetraparetic, limb weakness	Medulla	Cervical	Large-volume shunt, PFD, laminectomy, duraplasty	Alleviated	Not mentioned
	14, M	Vomiting, facial weakness, diplopia, hoarseness, ataxia	CN VI and VII palsies	Medulla, pons	Cervicothoracic	Suboccipital craniectomy, C-1 laminectomy, fenestration, duraplasty	Improved	4 yrs
Seki, T., 2004	27, M	Trunk numbness, limb weakness	Hyperreflexia, dysesthesia(R)	Medulla	C2-C7	PFD, laminectomy, duraplasty	Alleviated	5m
Aryan, H, 2004	55, M	Dysphagia, diplopia, gait difficulty	Bulbar, sensory, motor, and coordination deficits	Medulla	C1-T8	PFD, laminectomy, duraplasty, VP-shunt	No relief	3m
Lee, J., 2001	16, M	Dysphagia, drowsy	Quadriparesis	Medulla, pons	C1-T11	PFD, laminectomy, duraplasty, syringoperitoneal shunt	Remission	6m
Penarrocha, M., 2001	45, M	Orofacial pain	Hypoesthesia	Medulla	Cervical	Decompression, shunt	Resolved	2yrs
Galarza, M, 2001	9, M	Lethargy and respiratory arrest	Quadriparesis	Medulla	Cervical	Ventricle external drainage	Improved	Not mentioned
Nogues, M., 2000	58, F	Dysesthesia	Increased tone	Medulla	Cervicothoracic	None	None	None
Takahashi, Y., 1999	15, M	Gait disturbance, dysphagia	Horizontal nystagmus, ataxia, limb weakness	Medulla	Cervicothoracic	FMD, laminectomy	Improved	8yrs
Afifi, A, 1997	11, F	Diplopia, snoring	CN VI, XII palsies, arm hyporeflexia, leg hyperreflexia	Medulla, pons	Cervical	PFD, laminectomy, duraplasty, 4th-V shunt	Improved	6m
Anwer, U, 1996	47, F	Dysphagia, tough numbness	Hemiparalysis, Horner's syndrome	Medulla	Cervicothoracic	PFD	Improved	2m
Kanev, P, 1994	13, F	Diplopia	Diplopia	Medulla, pons	Cervical	PFD, laminectomy	Improved	2m
Rhoton, E. L., 1991	69, F	Limbs weakness and numbness	Hemiparesis, dysesthesia, hyperreflexia	Brain stem, syringocephaly	C2-C6	craniectomy, laminectomy, 4th V shunt	Resolved	Not mentioned
Weissman, J., 1990	46, M	Arm weakness and Dysesthesia, dysarthria and dysphagia	Torsional nystagmus, vocal cord paralysis	Medulla	C2-T8	Not mentioned	Not mentioned	Not mentioned
Okada, S., 1989	10, F	Limbs weakness, dysesthesia	Hemiparesis, dysesthesia, hyperreflexia, CN V palsies	Brain stem, syringocephaly	C1-T10	Craniectomy, laminectomy, duraplasty	Improved	1m
Bresnan, M., 1987	17, F	limbs numbness, nausea, vomiting, diplopia, oscillopsia, dysarthria	Rotatory nystagmus, CN V-VII palsies	Medulla	C2-T4	Decompressed, stent shunt, duraplasty	Improved	1m

C: cervical, CN: cranial nerve, ETV: endoscopic third ventriculostomy, F: female, FMD: foramen magnum decompression, ICP: intracranial pressure, M: male, m: month(s), PDF: posterior foramen decompression, SB: syringobulbia, SM: syringomyelia, T: thoracic, yrs: year(s), and VP: ventriculoperitoneal.

## Supplementary Materials

Supplementary Materials The supplementary material is complementary to Table 1. It is a summary of three case series of CM-I patients with syringobulbia [1, 5, 6].
