# Supplementary material for: Syringobulbia in Patients with Chiari Malformation Type I: A Systematic Review
Source: Biomed Res Int. 2019 Mar 19;2019:4829102. doi: 10.1155/2019/4829102 (PMC6444244; doi:10.1155/2019/4829102)
Supplement: Supplementary Materials — The supplementary material is complementary to Table 1. It is a summary of three case series of CM-I patients with syringobulbia [1, 5, 6]. [file 4829102.f1.pdf]

Supplementary Table: summary of three extra case series of CM-I patients with syringobulbia.

| Author(s), Year | Old | Sex | Manifestations | Examination Findings | Extent of SB | Extent of SM | Operation | Outcome | Follow-Up |
|---|---|---|---|---|---|---|---|---|---|
| Menezes, A. H., 2018 | 12 | F | Limb weakness, diplopia, snoring | CN VI, IX, X, XII palsies, Hyperreflexia, ptosis, | Medulla-pons, midbrain, no V4 connection | Holocord | Gliotic tonsils, veil over FM | Resolved except tongue fascic | 20 yrs |
| | 12 | F | Headache, diplopia, limb weakness | Nystagmus, dysesthesia, hemiparesis, hyperreflexia | Medulla | Holocord | Transoral decompration, PFD | Resolved | 4 yrs |
| | 14 | F | Poor coordination; headache, dysphagia | Nystagmus; spastic throughout; CN IX & X palsies | Medulla (left) | Cervical | Transoral decompration, PFD; veil over FM | Persistent hyperreflexia | 12 yrs |
| | 15 | F | Headache, dysphagia, ataxia, numbness | CN V, IX, X palsies | Age 1 yr: none<br>Age 15 yrs: medulla | cervicothoracic | PFD, VP-shunt, Transoral decompression medulla, gliosis, FM closed | Mild ataxia | 15 yrs |
| | 17 | M | Headache, sleep apnea, arm pain & numbness | CN V, IX, X palsies, arm dysesthesia, thoracic scoliosis | Medulla (left) | Holocord | SB opened into floor of V4 | Resolved | 13 yrs |
| | 17 | M | Sleep apnea, kyphoscoliosis, face numb, ptosis | Horner’s syndrome, CN V, IX, & X palsy | Medulla | Holocord | Veil occluding V4 | Resolved | 20yrs |
| | 11 | F | headache; neck pain; incoordination; diplopia | Ataxia, CN IX & X palsies; hyperreflexia | Medulla | Cervical | Entire (rt) tonsil in V4 | Resolved | 18yrs |
| | 16 | F | Headache, scoliosis | Ataxia, CN IX & X palsies, dysesthesia, hyporeflexia | Medulla (left) | Holocord, side tract in medulla to V4 | Tonsils in V4; veil over FM | Improved | 12yrs |
| | 18 | M | Arm pain | CN IX & X palsies | Medulla; no V4 connection | Holocord | Right tonsil filled V4 | Resolved | 4 yrs |
| | 15 | M | Limb weakness and dysesthesia, headache | Horner’s syndrome, hyperreflexia | Whole brain stem, syringocephaly | Holocord | Severe scar over FM | Improved | 4yrs |

| | | | | | | | | | |
|---|---|---|---|---|---|---|---|---|---|
| | 18 | M | Sleep apnea; headache; neck pain; dysphagia dysarthria | Nystagmus, CN IX & X palsies, horner's syndrome, tough fasciculation; palatal myoclonus | Medulla (left), ventrally | Holocord | Rt tonsil filled V4; veil over FM | Improved | 6yrs |
| | 14 | F | Headache, diplopia; scoliosis; incoordination | CN IX & X palsies; nystagmus; scoliosis; hyperreflexia, | Medulla | Cervical | Veil over foramen magnum; PFD and O–C2 fusion | Resolved | 3yrs |
| | 16 | M | Headache, dysphagia | CN IX & X palsies; hyperreflexia, dysesthesia | VP shunt | holocord | VP shunt | Improved | 1yr |
| Greenlee, J., 2005 | 12 | F | Diplopia, snoring | L VI, IX, X, XII palsies; ptosis; hand intrinsic weakness; foot drop; hyperreflexia | Medulla-inferior pons | Holochord | Posterior fossa decompression, C-1 laminectomy, and duraplasty | Tongue fasciculations otherwise normal | 4.1 |
| | 12 | F | Diplopia, headache | Mild hemiparesis; nystagmus; hyperreflexia; C2 hypesthesia | Medulla | Holochord | Posterior fossa decompression, C-1 laminectomy, and duraplasty | Hyperreflexic lower extremities | 2.5 |
| | 14 | F | Gait instability, incoordination | Nystagmus; hyperreflexia | Medulla | Cervica | Posterior fossa decompression, C-1 laminectomy, and duraplasty | Hyperreflexic | 1.8 |
| | 15 | F | Head and neck pain, dysphagia, hand paresthesia, snoring | L IX, X palsies; hyperreflexia; hand weakness | Medulla | Cervical | Posterior fossa decompression, C-1 laminectomy, and duraplasty | Normal | 3.5 |
| | 17 | M | "Eyes bouncing," arm paresthesias, snoring, sleep apnea | CN V palsy; upper extremity hypesthesia; hyperreflexia; thoracic scoliosis | Medulla | Holochord | Posterior fossa decompression, C-1 laminectomy, and duraplasty | Normal | 3.2 |
| | 17 | M | Numbness L face, eye droop, snoring | Ptosis, enophthalmos; face, limb hypesthesia, hypalgesia; hyporeflexia; scoliosis | Medulla | Holochord | Posterior fossa decompression, C-1 laminectomy, and duraplasty | Improved | 4.1 |
| Botelho, R., | 38 | F | Left hemiparesthesia | Left hemiparesthesia suspended | None | cervicothoracic(C1-T8) | None | None | - |

| | | | | | | | | | |
|---|---|---|---|---|---|---|---|---|---|
| 2000 | | | | anesthesia T4-T8 | | | | | |
| | 39 | F | Spastic tetraparesis，atrophy of the hands，snoring | Suspended dissociated anesthesia C6-T1 | None | cervicothoracic(C2-T6) | None | None | - |
| | 34 | F | Cerebellar ataxia，dysphagia, snoring | Cerebellar ataxia, Dysphagia | None | None | None | None | - |
| | 45 | M | Vertigo, snoring | Nystagmus | None | cervicothoracic(C2-T4) | None | None | - |
| | 54 | M | Brachial paresis, snoring | Hemipalatoparesis, dysphagia | None | Cervical | None | None | - |
| | 42 | F | Sensory disturbance, snoring | Right arm paresthesia and paresis, left leg paresis, right arm hypoesthesia | None | None | None | None | - |
| | 31 | F | Atrophy of the hands, Snoring | Fasciculation of the hands, painful anesthesia in the lower limbs, sensitive dissociation between T1-T6 | None | cervicothoracic(C3-T6) | None | None | - |
| | 50 | M | Spastic tetraparesis, Snoring | Fasciculation of the upper limbs, neck, and tongue | None | None | None | None | - |
| | 34 | F | Atrophy of the left hand, burning hands, snoring | Burning hands, left-hand atrophy | None | cervicothoracic(C1-T6) | None | None | - |
| | 70 | F | Complete left paresis, snoring | Complete left paresis | None | None | None | None | - |
| | 59 | M | Anakusis, facial paresis, spastic tetraparesis, snoring | Anakusis, facial paresis, spastic tetraparesis | None | None | None | None | - |

C: cervical, CN: cranial nerve, ETV: endoscopic third ventriculostomy, F: female, FMD: foramen magnum decompression, ICP: intracranial pressure, M: male, m: month(s), O: occipital, PDF: posterior foramen decompression, SB: syringobulbia, SM: syringomyelia, T: thoracic, yr(s): year(s), VP: ventriculo-peritoneal.
